# Hospitalisation of people with dementia: evidence from English electronic health records from 2008 to 2016

**DOI:** 10.1007/s10654-019-00481-x

**Published:** 2019-01-16

**Authors:** Andrew Sommerlad, Gayan Perera, Christoph Mueller, Archana Singh-Manoux, Glyn Lewis, Robert Stewart, Gill Livingston

**Affiliations:** 1 Division of Psychiatry, University College London, 6th Floor, Maple House, 149 Tottenham Court Road, London, W1T 7NF UK; 2 Camden and Islington NHS Foundation Trust, London, UK; 3 Institute of Psychiatry, Psychology and Neuroscience, King’s College London, London, UK; 4 South London and Maudsley NHS Foundation Trust, London, UK; 5 INSERM U 1018, Epidemiology of Ageing and Age-related diseases, Villejuif, France; 6 Department of Epidemiology and Public Health, University College London, London, UK

**Keywords:** Dementia, Hospitalization, Health services, Prognosis, Geriatrics

## Abstract

Hospitalisation of people with dementia is associated with adverse outcomes and high costs. We aimed to examine general, i.e. non-psychiatric, hospitalisation rates, changes since 2008 and factors associated with admission. We also aimed to compare admission rates of people with dementia with age-matched people without dementia. We conducted a cohort study of adults ≥ 65 years, with dementia diagnosed during the 2008–2016 study window, derived from a large secondary mental healthcare database in South London, UK. We used national general hospital records to identify emergency and elective hospitalisations. We calculated the cumulative incidence and rate of hospitalisation and examined predictors of hospitalisation using negative binomial regression, with multiple imputation for missing covariate data. We calculated age-standardised admission ratio for people with dementia compared to those without. Of 10,137 people, 50.6% were admitted to hospital in the year following dementia diagnosis and 75.9% were admitted during median 2.5 years follow-up. Annual admission rate was 1.26/person-year of which 0.90/person-year were in emergency. Emergency hospitalisation rate increased throughout the study period. Compared to controls without diagnosed dementia in the catchment area, the age-standardised emergency admission ratio for people with dementia was 2.06 (95% CI 1.95, 2.18). Male, older, white and socio-economically deprived people and those with clinically significant comorbid physical illness, depressed mood, activity of daily living or living condition problems had more hospitalisations. Emergency hospitalisations of people with dementia are higher than those without, and increasing. Many factors associated with admission are social and psychological, and may be targets for future interventions that aim to reduce avoidable admissions.

**Electronic supplementary material:**

The online version of this article (10.1007/s10654-019-00481-x) contains supplementary material, which is available to authorized users.

## Introduction

Although age-specific incidence of dementia is falling in the US [1] and UK [2], overall numbers affected are rising due to population ageing [2]. Management of people with dementia in general hospitals is challenging as they often have neuropsychiatric symptoms [3], multi-morbidity [4], and difficulty engaging with management plans [5]. People with dementia receive more antipsychotics and sedatives [6], have longer and costlier hospital admissions [7, 8], and often decline during admission [9]. Therefore reducing admissions, many of which may be avoidable [10], would be advantageous for the patient and care-provider. Understanding the correlates of admission of people with dementia would help us to understand the factors leading to admission.

Previous studies examining hospitalisation in dementia have used research samples which under-represent [11, 12] or exclude [13, 14] more severe dementia or physical illness so have limited generalisability. Other studies have been small [13–18], with short follow-up [7, 13, 19, 20]. Many have ascertained admission information from carers [13, 14] resulting in recall bias, or local hospital registers with limited generalisability [15, 19, 21]. No previous study has examined hospitalisation trends, yet there has been increased focus on improving interventions for dementia in recent years [22]. Our study includes all people with dementia within a large secondary healthcare service including memory clinics, the principal diagnostic services to which people with suspected dementia are referred [23], therefore is representative of people with dementia, and uses national hospitalisation register data.

We aimed to:

1. describe general hospital admission rates within the national health care provider in people with dementia diagnosed in secondary mental healthcare services
2. compare admission rates with an age-standardised control population without dementia
3. identify factors associated with hospitalisation, including time trends between 2008 and 2016.

## Methods

### Study design and data sources

We conducted a cohort study using two linked clinical datasets, described below.

The South London and Maudsley (SLaM) National Health Service (NHS) Foundation Trust Case Register “Clinical Record Interactive Search” (CRIS) data extraction tool.

We used the CRIS resource [24] to identify dementia cases for our cohort. It provides research access to anonymised electronic medical records from SLaM, which provides mental healthcare including dementia assessment and management [25] for a London, UK, catchment area containing 1.2 million residents. CRIS enables anonymised data extraction from structured record fields, and unstructured text data using natural language processing algorithms [24, 26] developed using General Architecture for Text Engineering (GATE) software [27].

The Oxfordshire Research Ethics Committee C (reference 08/H0606/71 + 5) approved use of the data resources for secondary analysis.

NHS Digital Hospital Episode Statistics (HES).

We collected hospitalisation data from HES, which records all English NHS hospitalisation data collected by hospital providers [28]. We used records of general (non-psychiatric) admissions, identified with codes for emergency (unplanned) and elective (planned, e.g. for surgery, renal dialysis, chemotherapy) admissions. At the time of analysis, data were available until 31 March 2016.

### Study participants

We retrieved records from CRIS of all patients aged ≥ 65 years who had a diagnosis of dementia entered for the first time on their electronic medical record during the study window from 1 January 2008 to 31 March 2015. We excluded patients whose first electronic record of dementia preceded 2008, as we aimed to include those with newly-diagnosed dementia and patients first diagnosed after March 2015 to ensure all had at least 1 year potential HES follow-up.

We derived dementia status using either structured ICD-10 [29] diagnosis fields (codes F00x-F03x) or unstructured text, using a GATE-derived algorithm, which has been found to have precision 99% and recall 98% for dementia diagnosis [24]. Of the 10,137 patients with dementia, 2970 (29.3%) were ascertained using GATE alone, with similar characteristics to those with ICD-10 diagnosis (eTable 1).

Hospitalisation data for people with dementia were generated by linking people with dementia diagnosed in CRIS to HES admission data; we retrieved the dates of each hospitalisation after the first CRIS-recorded dementia diagnosis until death or 31 March 2016. A control dataset included HES admission data for all other residents of the catchment area, without dementia diagnosis. These data only include people with ≥ 1 admission, so we used the 2011 national census data [30] on over-65s in the catchment area to ascertain the denominator population.

### Covariates

We extracted data from CRIS on age, sex, ethnicity, marital status, and socioeconomic status estimated using the Index of Multiple Deprivation (IMD) [31]; a higher score indicates more socioeconomic deprivation. We extracted dementia sub-type at last recording (grouped as Alzheimer’s, vascular, Lewy body, other or unspecified (where aetiology unrecorded).) We estimated dementia severity using Mini Mental State Examination (MMSE) [32] scores (from structured and unstructured fields). For other aspects of clinical presentation, we used the Health of the Nation Outcome Scales (HoNoS), a 12-domain clinician-rated instrument completed at first assessment. It comprises subscales rated 0 (no problem) to 4 (severe/very severe problem) and has acceptable/good psychometric properties [33]. We dichotomised scores: 0 and 1 were grouped as no/minor problems, scores of ≥ 2 represented clinically significant problems. We used eight HoNOS domains of interest, rating problems with: agitation, self-injury, substance use, physical illness, hallucinations, depressed mood, activities of daily living, or living conditions. All covariates were taken from the recording closest to dementia diagnosis, except for dementia subtype, for which we used the last recording.

### Statistical analysis

We first described the characteristics of our sample and then compared these according to hospitalisation during the study window.

### Cumulative incidence and admission rate of hospitalisation

We calculated the cumulative incidence of hospitalisation (= number of people admitted at least once during study window/total number in the cohort) and the admission rate (= all admissions/person-years (PY), calculated as time between CRIS dementia diagnosis, and death or end of study window), with 95% confidence intervals [34]. We examined these outcomes for all admissions, then those coded as emergency and elective, during the first year following diagnosis and all follow-up. We then determined the distribution of the count of hospitalisation.

### Age-standardised admission ratio for dementia

We calculated the age-standardised admission ratio for emergency and elective admissions (ratio of observed admissions for people with dementia to the expected admissions based on the control population, standardised to the control age distribution) [34]. We examined admissions during 2011, as the control denominator population was taken from the 2011 census. We used 5 year age bands for standardisation and calculated the control population by excluding people with dementia diagnosed between 2008 and 2016 from the control dataset and subtracting these from the denominator population.

### Association of sociodemographic and clinical factors with hospitalisation

We used negative binomial regression to analyse associations of sociodemographic and clinical factors with the number of emergency and elective hospitalisations. We included in our multivariable analysis age, sex, marital status, ethnicity, IMD, MMSE, dementia subtype, HoNOS domains and year of diagnosis as a categorical variable, and in a separate analysis, as an ordinal variable. We included time of follow-up in our model as an exposure variable.

Our primary analyses examined predictors of admissions during the first year after diagnosis, as covariates were taken from time closest to diagnosis so held more salience for proximal admissions. We also judged that assessing admission rates by year of diagnosis would be biased if we used the full study period as, despite adjusting for years of follow up, those with longer duration of follow-up would be older when studied which could affect admission risk. In sensitivity analyses, we analysed predictors of hospital admissions throughout the full study period.

As 22% of the cohort had missing data on at least one predictor, we also conducted sensitivity analyses using multiple imputation by chained equations [35] for missing covariates to maximise statistical power. We used the *mi* package in STATA to create five imputed datasets constructed from all potential covariate and outcome variables, before using negative binomial regression on each imputed dataset and Rubin’s rules [36] to combine coefficients.

We considered whether admission rates may be affected by more physically unwell people being diagnosed by the increasingly common liaison/consultation psychiatry services [37]. We therefore conducted post hoc sensitivity analyses of admission cumulative incidence and rate and the association of number of admission with year of diagnosis, while excluding people whose diagnosis was within 1 month of consultation psychiatry assessment.

## Results

We obtained data on 10,137 eligible adults with dementia aged ≥ 65 years. The characteristics of the sample at dementia diagnosis are summarised in Table 1. The mean age of people was 82.1 (standard deviation (SD) 7.2) years. The majority were female and white, with African/Caribbean forming the largest minority ethnic group. Mean MMSE score was 18.6 (SD 6.3) and around half of the cohort had Alzheimer’s disease.

**Table 1:** Socio-demographic and clinical characteristics of all people with dementia, and according to whether admitted to general hospital during follow-up

Characteristic		All people with dementia (n = 10,137)		Admitted to hospital (n = 7693)		Not admitted to hospital (n = 2444)		Significance test^a^
		n	%	n	*%*	n	%	
Age at diagnosis	Mean (SD)	82.1 (7.2)		82.1 (7.0)		81.8 (7.7)		t = − 2.3, *p* = 0.02
	65–69	600	5.9	412	5.4	188	7.7	χ^2^ = 47.4, *p* < 0.001
	70–74	1203	11.9	866	11.3	337	13.8	
	75–79	1998	19.7	1541	20.0	457	18.7	
	80–84	2602	25.7	2044	26.6	558	22.8	
	85–89	2446	24.1	1890	24.6	556	22.8	
	90 +	1288	12.7	940	12.2	348	14.2	
	Missing	0		0		0		
Sex	Female	6262	61.8	4662	60.6	1600	65.5	χ^2^ = 18.5, *p* < 0.001
	Missing	1		1		0		
Ethnicity	White	7640	77.3	5915	78.4	1725	73.8	χ^2^ = 28.9, *p* < 0.001
	Asian	453	4.6	345	4.6	108	4.6	
	African/Caribbean	1445	14.6	1050	13.9	395	16.9	
	Other	344	3.5	234	3.1	110	4.7	
	Missing	255		149		106		
Marital status^b^	Married	3202	33.5	2454	33.5	748	33.4	χ^2^ = 4.5, *p* = 0.21
	Divorced	769	8.0	590	8.1	179	8.0	
	Widowed	3892	40.7	3007	41.1	885	39.5	
	Single	1701	17.8	1270	17.4	431	19.2	
	Missing	573		372		201		
Mean deprivation score^b^ (SD)		27. 2 (11.1)		27.3 (11.1)		26.7 (11.1)		t = − 2.14, *p* = 0.03
	Missing	40		22		18		
Mean MMSE^b^ (SD)		18.6 (6.3)		18.6 (6.2)		18.6 (6.5)		t = − 0.17, *p* = 0.87
	Missing	1579		1049		530		
Problem^b^ with: (from HoNOS subscale)	Agitation	1998	20.7	1494	20.2	504	22.2	χ^2^ = 4.3, *p* = 0.04
	Self-injury	136	1.4	108	1.5	28	1.2	χ^2^ = 0.65, *p* = 0.42
	Alcohol/drugs	302	3.1	233	3.2	69	3.0	χ^2^ = 0.07, *p* = 0.79
	Physical illness	5511	57.1	4307	58.3	1204	53.1	χ^2^ = 18.9, *p* < 0.001
	Hallucinations	1354	14.1	1058	14.4	296	13.1	χ^2^ = 2.2, *p* = 0.14
	Depressed mood	1416	14.7	1083	14.7	333	14.7	χ^2^ = 0.002, *p* = 0.96
	Daily living	5981	62.1	4601	62.5	1380	61.0	χ^2^ = 1.75, *p* = 0.19
	Living conditions	1220	12.8	952	13.1	268	11.9	χ^2^ = 1.9, *p* = 0.17
	Missing^c^	513		452		315		
Last recorded dementia diagnosis	Alzheimer’s disease	5166	51.0	3884	50.5	1282	52.5	χ^2^ = 13.8, *p* = 0.008
	Vascular dementia	2223	21.9	1741	22.6	482	19.7	
	Lewy body dementia	299	2.9	237	3.1	62	2.5	
	Other dementia	691	6.8	529	6.9	162	6.6	
	Unspecified dementia	1758	17.3	1302	16.9	456	18.7	
Year of diagnosis	2008	1215		1015	83.5	200	16.5	χ^2^ = 371.8, *p* < 0.001
	2009	1177		970	82.4	207	17.6	
	2010	1346		1094	81.3	252	18.7	
	2011	1445		1162	80.4	283	19.6	
	2012	1476		1131	76.6	345	23.4	
	2013	1545		1153	74.6	392	25.4	
	2014	1515		944	62.3	571	37.7	
	2015	418		224	53.6	194	46.4	

*HoNOS* health of the Nation Outcome Scale, *MMSE* mini-mental state examination

^**a**^Chi square test used to compare characteristics between admitted and non-admitted groups for categorical variables and t test used for continuous variables

^b^Based on clinical assessment nearest to first dementia diagnosis

^c^Figure for missing HoNOS score is for the HoNOS domain with most missing information

### Cumulative incidence and admission rate of hospitalisation

**Fig. 1:**
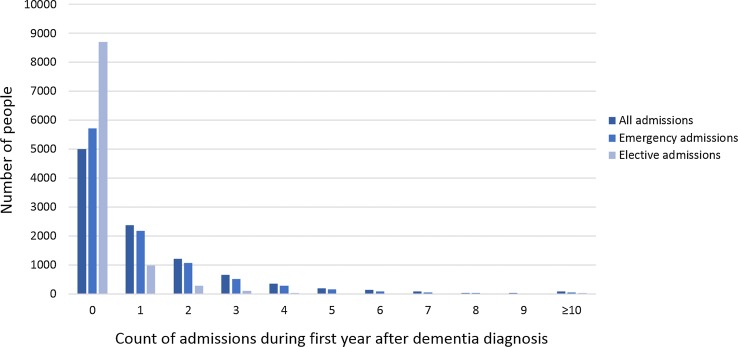
Distribution of count of general hospital admissions for people with dementia during first year after diagnosis (n = 10,137)

During the first year following dementia diagnosis, 5127 [50.6% (95% CI 49.6, 51.6)] were admitted to hospital. The hospitalisation rate during the first year after diagnosis was 1.05/PY (1.03, 1.07) for emergency admissions and 0.44/PY (0.43, 0.46) for elective admissions. hospitalisation count distribution are shown in Fig. 1 (full data in eTable 2); 2245 people (22.2%) had ≥ 2 and 41 (0.4%) of the sample had ≥ 10 emergency hospitalisations during the year after diagnosis.

During the study window (median 2.5 years; interquartile range 1.3, 4.1; maximum 8.2 years), 7693 [75.9% (75.0, 76.7)] (Table 2) were hospitalized. During 28,425.3 PY total follow-up, the cohort experienced 35,716 general hospital admissions, of which 25,634 (71.8%) were emergency. The hospitalisation rate was 1.26/PY (1.24, 1.27), of which 0.90/PY (0.89, 0.91) were emergency, and 0.35/PY (0.35, 0.36) were elective.

**Table 2:** Rate of general hospital admissions of people with dementia 2008–2016

Number of people ever admitted	7693/10,137
Cumulative incidence of any admission (%) (95% confidence interval)	75.9 (75.0, 76.7)
Person years^a^	28,425.3
Total number of all admissions	35,716
Admission rate (/PY) (95% confidence interval)	1.26 (1.24,1.27)
Number of emergency^b^ admissions	25,634
Emergency admission rate (/PY)	0.90 (0.89, 0.91)
Number of elective^b^ admissions	10,082
Elective admission rate (/PY) (95% confidence interval)	0.35 (0.35, 0.36)

*PY* person years

^a^PY calculated based on time between initial dementia diagnosis AND death OR end of window (whichever was earliest)

^b^Elective/emergency admission status according to admission record coding

In our sensitivity analysis excluding 1293 people whose dementia diagnosis was within 1 month of consultation psychiatry assessment, 76.1% (75.2, 77.0) of the remaining 8844 were admitted during the study window; hospitalisation rate = 1.13/PY (1.12, 1.14), of which 0.86 (0.84, 0.87) were emergency.

### Age-standardised admission ratio for dementia

The control group consisted of 105,889 residents without dementia diagnosis from SLaM, who had 31,233 emergency admissions and 62,796 elective admissions during 2011 (Table 3). The age-standardised admission ratio for people with dementia compared to those without was 2.06 (1.95, 2.18) for emergency admissions and 1.00 (0.93, 1.07) for elective admissions.

**Table 3:** Standardised admission ratio for people with dementia compared to those without diagnosed dementia, during 2011

	Age-groups	People without dementia			People with dementia			Expected number of admissions	
		n	Number of admissions	Admission rate (/yr)	n	Number of admissions	Admission rate (/yr)		
Emergency admissions	65–69	32,441	5146	0.16	76	81	1.07	12.1	Standardised emergency admission ratio (= observed/ expected × 100)
	70–74	27,148	5962	0.22	186	145	0.78	40.8	
	75–79	20,744	6199	0.30	300	254	0.85	89.7	
	80–84	14,178	6209	0.44	380	324	0.85	166.4	
	85–89	7591	4649	0.61	331	340	1.03	202.7	
	90 +	3787	3068	0.81	172	199	1.16	139.3	
	Total	105,889	31,233		1445	1343		651	2.06 (1.95, 2.18)
Elective admissions	65–69	32,441	16,938	0.52	76	41	0.54	39.7	Standardised elective admission ratio (= observed/expected × 100)
	70–74	27,148	16,772	0.62	186	206	1.11	114.9	
	75–79	20,744	14,622	0.70	300	305	1.02	211.5	
	80–84	14,178	9931	0.70	380	189	0.50	266.2	
	85–89	7591	3668	0.48	331	62	0.19	159.9	
	90 +	3787	865	0.23	172	25	0.15	39.3	
	Total	105,889	62,796		1445	828		831	1.00 (0.93, 1.07)

## Association of sociodemographic and clinical factors with hospitalisation

### Emergency hospitalisation

Emergency hospitalisation rate within the first year after diagnosis (Table 4) was higher in fully-adjusted models for those who were older, from a more socio-economically deprived area, rated as having problem with physical illness, depressed mood, activities of daily living, or their living conditions, and those with non-Alzheimer’s dementias. Women and people from minority ethnic groups had lower emergency hospitalisation rates.

**Table 4:** Predictors of general hospital admissions during first year after dementia diagnosis; multivariable negative binomial regression (n = 7863 with complete covariate data)

Characteristic		Emergency hospital admissions		Elective hospital admissions	
		IRR (95% CI)	*p* value	IRR (95% CI)	*p* value
Age (per 1 year increment)		**1.03 (1.02, 1.03)**	<** 0.001**	**0.96 (0.95, 0.98)**	<** 0.001**
Sex	Female	**0.77 (0.71, 0.84)**	<** 0.001**	**0.58 (0.49, 0.70)**	<** 0.001**
Ethnicity	White (Ref.)	1		1	
	Asian	**0.79 (0.66, 0.96)**	**0.02**	1.23 (0.85, 1.77)	0.27
	African/Caribbean	**0.80 (0.72, 0.90)**	<** 0.001**	**1.43 (1.13, 1.81)**	**0 003**
	Other	**0.69 (0.55, 0.87)**	**0.001**	1.24 (0.79, 1.94)	0.35
Marital status	Married (Ref.)	1		1	
	Divorced	1.13 (0.97, 1.30)	0.11	1.33 (0.98, 1.82)	0.07
	Widowed	1.10 (1.00, 1.21)	0.05	1.03 (0.84, 1.27)	0.79
	Single	1.10 (0.98, 1.23)	0.11	0.95 (0.75, 1.21)	0.68
Deprivation score (per 10-unit increase in deprivation)		**1.05 (1.01, 1.09)**	**0.006**	**0.92 (0.86, 0.99)**	**0.04**
MMSE (per 1 unit decrease)		1.01 (1.00, 1.01)	0.05	**0.95 (0.94, 0.97)**	<** 0.001**
Problem with (from HoNOS subscale)^a^:	Agitated behaviour	1.02 (0.92, 1.13)	0.72	0.93 (0.74, 1.17)	0.56
	Self-injury	1.24 (0.92, 1.68)	0.16	0.64 (0.30, 1.36)	0.24
	Problem-drink/drugs	1.12 (0.91, 1.38)	0.30	1.43 (0.92, 2.23)	0.11
	Physical illness	**1.73 (1.59, 1.88)**	<** 0.001**	**1.81 (1.51, 2.18)**	<** 0.001**
	Hallucinations	1.03 (0.92, 1.15)	0.56	0.84 (0.66, 1.06)	0.14
	Depressed mood	**1.14 (1.02, 1.26)**	**0.02**	**0.67 (0.53, 0.85)**	**0.001**
	Daily living	**1.18 (1.08, 1.28)**	<** 0.001**	**1.33 (1.10, 1.62)**	**0.004**
	Living conditions	**1.16 (1.04, 1.30)**	**0.008**	0.82 (0.64, 1.05)	0.11
Last recorded dementia diagnosis	Alzheimer’s disease (Ref.)	1		1	
	Vascular dementia	**1.41 (1.28, 1.55)**	<** 0.001**	**2.18 (1.75, 2.72)**	<** 0.001**
	Lewy body dementia	1.18 (0.95, 1.47)	0.13	1.46 (0.93, 2.29)	0.10
	Other dementia	1.08 (0.93, 1.25)	0.33	1.17 (0.84, 1.62)	0.35
	Unspecified dementia	**1.55 (1.40, 1.73)**	<** 0.001**	**1.43 (1.11, 1.83)**	**0.005**
Year of diagnosis (per 1 year later)	2008 (Ref.)	1		1	
	2009	**1.18 (1.01, 1.38)**		0.99 (0.71, 1.39)	
	2010	**1.26 (1.08, 1.47)**		1.37 (0.99, 1.90)	
	2011	**1.21 (1.04, 1.41)**		**1.78 (1.30, 2.43)**	
	2012	**1.32 (1.14, 1.53)**		**1.54 (1.13, 2.11)**	
	2013	**1.28 (1.10, 1.48)**		**1.50 (1.09, 2.06)**	
	2014	**1.29 (1.11, 1.50)**		1.34 (0.97, 1.84)	
	2015	**1.39 (1.12, 1.73)**		0.91 (0.56, 1.47)	
	Per one year later	**1.03 (1.01, 1.05)**	**0.001**	1.04 (1.00, 1.08)	0.07

*CI* confidence Interval, *HoNOS* health of the nation outcome scales, *IRR* incidence rate ratio, *MMSE* mini-mental state examination

Bold figures indicate *p* < 0.05 in multivariable analysis

^a^HoNOS subscale, dichotomised to 0–1 (no or minor problem) and 2–4 (problem behaviour)

Hospitalisation rates increased over time; IRR for people diagnosed in 2015, compared to 2008 = 1.39 (1.12, 1.73). Applying year of diagnosis as an ordinal independent variable, the IRR for each year increment was 1.03 (1.01, 1.05).

In sensitivity analyses, results were similar when we analysed the full study period (*eTable 3*) except that the association with year of diagnosis was attenuated. We found similar results when using multiple imputation for missing covariates (*eTable* *4*). Excluding people diagnosed during consultation psychiatry assessment did not change our results.

### Elective hospitalisation

Elective hospitalisation rates (Table 4) were higher for those who were younger, African/Caribbean ethnicity, from less socio-economically deprived areas, and those who had better cognition, problem with physical illness, activities of daily living and non-Alzheimer’s dementias. Women and those with depressed mood at diagnosis had lower Hospitalisation rates. Elective admission rates did not change during the study period. Results were consistent in our sensitivity analyses (appendices 2,3).

## Discussion

In a large secondary care cohort, we found high and increasing rates of emergency hospitalisation for people with dementia. Half of people with dementia were admitted to hospital in the year after diagnosis, three quarters were admitted during 2.5 years median follow-up, and multiple admissions were common. The emergency admission rate was 0.90 per person year and people with dementia had 2.1 times more emergency admissions than age-matched controls without diagnosed dementia; elective admissions did not differ between people with dementia and those without. Emergency but not elective hospitalisation rates increased since 2008. We found higher emergency hospitalisation rates in people who were older, male, white, more socio-economically deprived people and those with non-Alzheimer’s dementia, worse activities of daily living and problems with their living conditions, and physical illness or depressed mood at diagnosis. A different pattern of predictors was found for elective admissions which were more frequent with younger age, African/Caribbean ethnicity, less socio-economic deprivation and milder dementia.

The hospitalisation rate in our study is higher than that reported in any previous study. Specifically, US research cohorts have reported rates of only 0.16/PY admissions between 1991 and 2006 [17] and 0.42/PY admissions during 1994–2007 [16], and a French cohort had 0.22/PY admissions during 2000–2004 [14]. This study’s proportion admitted during 1 year (56%) is also higher than any other study reporting this outcome, whose estimates were between 24 and 41% [7, 13, 19, 20, 38]. Admission rates for people with dementia compared to those without was higher in our study than in three of the four previous US studies examining this [7, 16, 21]. Direct comparison of admissions between countries is difficult because healthcare service organisation differ, but admission rates for the general populations of the UK and US have been reported as similar [39].

The higher rate in our study is partly due to our sample being derived from a clinical sample in which no-one with dementia was excluded and our use of national hospital records ensuring that almost complete outcome data—1% of UK hospital services are non-NHS [40] and this figure would be lower for emergency admission of people with dementia—compared to population-based cohorts prone to ‘healthy volunteer’ selection bias [11] and selective attrition of more unwell participants [12]. Our cohort was also older at baseline (mean 82 years) than those in the other studies (mean 76–78 years). Furthermore, our study uses more recent data than any other study—we found increasing admissions from 2008 to 2016, so recent data would show higher hospitalisation rates. Increasing UK hospitalisation rates for older people generally have been reported by the Kings Fund [41], due to greater multi-morbidity related to longevity [42], lower tolerance of risk by public and professionals [43], and reduced availability of community services. Dementia diagnosis in community settings [44] and hospitals [45] has increased over the past decade and the 2012 UK policy of seeking possible dementia cases in elderly hospital inpatients [46] and referring to memory services for diagnosis may have resulted in more physically unwell people being diagnosed in the later years of our study, though our findings were adjusted for physical illness severity. While excluding people diagnosed by consultation psychiatry gave slightly lower admission rates, the role of these services did not explain changing admission rates over time.

We found as expected that having comorbid physical illness at diagnosis was the strongest predictor of subsequent emergency and elective hospitalisation. Previous studies have indicated that more severe dementia is associated with higher admission rates [15] and we found that functional impairment, rather than cognitive impairment, is independently associated with emergency and elective admission, consistent with two previous studies [13, 14]. Our findings that men [17, 47], lower socio-economic groups [13], non-Alzheimer’s dementias [16, 19], and depression [14, 18] are associated with emergency admission are also consistent with previous studies. This research, from a more ethnically diverse area than previous studies, adds that non-White ethnic groups have fewer emergency admissions, possibly a result of greater family care [48]. We also found that problematic living conditions, reflecting whether home support is sufficient to meet basic necessities of light, heat and hygiene [49], was associated with emergency hospitalisation. This finding and the association with ethnicity and socio-economic status suggests that social factors are important predictors of admission risk. Declining UK social care spending since 2010 has been reported despite increasing numbers of older people [50] and UK social care has recently been described as ‘struggling to meet the needs of older people’ [51]. Our findings support the impression that social care pressures have resulted in insufficient home care and increasing hospitalisations.

We identified a different pattern of predictors for elective hospitalisations. Younger and less cognitively impaired people had higher rates of elective admission, suggesting that planned treatment is preferentially delivered to those with less advanced dementia. Less socio-economically deprived people had more elective admissions, which may reflect more healthcare engagement in these groups or bias in clinician decision making [52]. African/Caribbean people had higher admission rates, which may be due to higher rates of elective renal dialysis [53], which have been found to be 1.88 times higher in the black UK population compared to white ethnic groups [54].

### Limitations

Our study has potential limitations. Analyses of hospitalisation predictors were limited to those recorded during routine practice and data on physical comorbidity were limited; we used the HoNOS physical illness domain in our analyses which has been reported to have acceptable psychometric properties [33] and its strong association with admission in our study supports its predictive validity. However, future studies using high quality data on premorbid illness should consider which conditions are associated with hospitalisation in order to identify specific targets for preventative medical treatment. We did not have data on other potentially important factors, such as influenza or pneumococcal vaccination status or residential setting. One study found that admission rates of nursing home residents were one quarter that of the community-dwelling population with dementia [55], suggesting that care home residence may protect against hospitalisation in people with severe dementia. However, our analysis studied admission during the first year after diagnosis, so the proportion of patients living in nursing homes is likely to be considerably lower than the overall UK figure of 16% of people with dementia estimated to live in nursing homes [56].

CRIS only contains records of people with dementia who have consulted secondary healthcare services so our results may not generalise to those with undiagnosed dementia. Diagnosis of dementia may indicate those with more active health-seeking behaviours, possibly including hospitalisation, thereby overestimating hospitalisation rate of all people with dementia whether diagnosed or undiagnosed. However, a recent systematic review found no correlation between care-seeking behaviours and hospitalisation rate [57], so care seeking behaviour is unlikely to affect the generalisability of our results.

Our study may also not generalise to those whose dementia was diagnosed in primary care or geriatric medicine settings, which is the norm in some areas of the UK [58]. However, this is relatively rare in the studied catchment area, where the custom is to refer to memory services as these are the mainstay of UK dementia diagnostic practice. This secondary mental healthcare service also provides some post-diagnostic care, so individuals diagnosed in other services may have subsequently received CRIS diagnosis. In our previous study, *92% of people with dementia recorded in hospital records had previously been seen by the mental health services and given a diagnosis of dementia in CRIS* [45]. We therefore judge our study sample to encompass and closely resemble most of those with diagnosed dementia in the large inner-city and suburban catchment area. In addition, our analyses use data from a single mental healthcare provider whose services may differ from other providers, so the analysed sample may not generalise nationally. However the estimated proportion of people with dementia living in the catchment area who have been diagnosed is relatively high at 75.2%, compared to 67.6% nationally [59]. Finally, some of the control population in our analysis may have had undiagnosed dementia, which would mean that our study underestimates the standardised admission ratio as people with undiagnosed dementia may have more admissions than those without dementia.

## Conclusions

The current study provides evidence for high and rising emergency hospitalisation rates of people with dementia. Hospital admission of people with dementia can be harmful and costly and, while we recognise that many admissions are appropriate, the potentially modifiable factors associated with admission in this study suggest that many are, in all probability, avoidable such as by improving quality of living conditions and maximising functional ability. Developing effective interventions to reduce avoidable admissions of community-dwelling people with dementia is a major priority; a recent systematic review found no effective interventions [60] although there has been success in those without dementia [61]. Understanding the causes of admissions informs the development of strategies to reduce admission, allowing future evaluation in trials. That admission rates are rising could indicate that cross-specialty health and social care is currently not meeting the needs of people with dementia and their carers [50, 51]. Reducing expensive, potentially harmful hospitalisation can only be done by improving alternatives in the community.

## Electronic supplementary material

Below is the link to the electronic supplementary material.

Supplementary material 1 (DOCX 59 kb)
