# Supplementary material for: Hospitalisation of people with dementia: evidence from English electronic health records from 2008 to 2016
Source: Eur J Epidemiol. 2019 Jan 16;34(6):567–77. doi: 10.1007/s10654-019-00481-x (PMC6497615; doi:10.1007/s10654-019-00481-x)
Supplement: Supplementary file 1 — Supplementary material 1 (DOCX 59 kb) [file 10654_2019_481_MOESM1_ESM.docx]

Online only supplements:

**eTable 1: Baseline characteristics of people with dementia ascertained from structured diagnostic field and those ascertained using the natural language programming application (n=10,137)**

**eTable 2: Distribution of count of general hospital admissions of people with dementia (n=10,137)**

**eTable 3: Predictors of general hospital admissions of people with dementia during all follow-up: multivariable negative binomial regression (n=7,863 with complete covariate information).**

**eTable 4: Predictors of general hospital admissions during first year after dementia diagnosis; multivariable negative binomial regression using multiple imputation for missing covariates (n=10,137).**

**Appendix 1: Baseline characteristics of people with dementia ascertained from structured diagnostic field and those ascertained using the natural language programming application (n=10,137)**

| Characteristic | | People with dementia  (n=10,137) | |  | Diagnosis in structured field  (n=7,167) | | Diagnosis by NLP algorithm  (n=2,970) | | Significance test ^a^ |
| --- | --- | --- | --- | --- | --- | --- | --- | --- | --- |
|  |  | **n** | **%** |  | **n** | ***%*** | **n** | **%** |  |
| Mean age at diagnosis (S.D.) | | 82.1 (7.2) | |  | 82.3 (7.1) | | 81.4 (7.4) | | t=6.1, p < 0.001 |
|  | *Missing* | *0* | |  | *0* | | *0* | |  |
| Sex | Female | 6262 | 61.8 |  | 4426 | 61.8 | 1836 | 61.8 | χ^2^ = 2.4, p = 0.30 |
|  | *Missing* | *1* | |  | *0* | | *1* | |  |
| Ethnicity | White | 7640 | 77.3 |  | 5403 | 77.7 | 2237 | 76.3 | χ^2^ = 26.7, p < 0.001 |
|  | Asian | 453 | 4.6 |  | 318 | 4.6 | 135 | 4.6 |  |
|  | African/Caribbean | 1445 | 14.6 |  | 1001 | 14.4 | 444 | 15.2 |  |
|  | Other | 344 | 3.5 |  | 230 | 3.3 | 114 | 3.9 |  |
|  | *Missing* | *255* | |  | *215* | | *40* | |  |
| Marital status ^b^ | Married | 3202 | 33.5 |  | 2208 | 32.9 | 994 | 34.9 | χ^2^ = 25.7, p < 0.001 |
|  | Divorced | 769 | 8.0 |  | 543 | 8.1 | 179 | 6.3 |  |
|  | Widowed | 3892 | 40.7 |  | 2757 | 41.1 | 1135 | 39.8 |  |
|  | Single | 1701 | 17.8 |  | 1204 | 17.9 | 497 | 17.4 |  |
|  | *Missing* | *573* | |  | *455* | | *118* | |  |
| Mean deprivation score ^b^ (S.D.) | | 27. 2 (11.1) | |  | 27.0 (11.3) | | 27.7 (10.7) | | t=-3.1, p = 0.002 |
|  | *Missing* | *40* | |  | *25* | | *15* | |  |
| Mean MMSE ^b^ (S.D.) | | 18.6 (6.3) | |  | 18.4 (6.3) | | 19.2 (6.3) | | t=-5.4, p < 0.001 |
|  | *Missing* | *1579* | |  | *1200* | | *379* | |  |
| Problem ^b^ with:  (from HoNOS subscale) | Agitation | 1998 | 20.7 |  | 1408 | 19.7 | 590 | 19.9 | χ^2^ = 11.4, p = 0.003 |
|  | Self-injury | 136 | 1.4 |  | 84 | 1.2 | 52 | 1.8 | χ^2^ = 15.7, p < 0.001 |
|  | Alcohol / drugs | 302 | 3.1 |  | 208 | 2.9 | 94 | 3.2 | χ^2^ = 11.3, p = 0.004 |
|  | Physical illness | 5511 | 57.1 |  | 3905 | 54.5 | 1606 | 54.1 | χ^2^ = 12.8, p = 0.002 |
|  | Hallucinations | 1354 | 14.1 |  | 902 | 12.6 | 452 | 15.2 | χ^2^ = 24.9, p < 0.001 |
|  | Depressed mood | 1416 | 14.7 |  | 930 | 13.0 | 486 | 16.4 | χ^2^ = 28.6, p < 0.001 |
|  | Daily living | 5981 | 62.1 |  | 4254 | 59.4 | 1727 | 58.2 | χ^2^ = 20.1 p < 0.001 |
|  | Living conditions | 1220 | 12.8 |  | 825 | 11.5 | 395 | 13.3 | χ^2^ = 30.1, p < 0.001 |
|  | *Missing ^c^* | *605* | |  | *483* | | *122* | |  |
| Last recorded dementia diagnosis | Alzheimer’s Disease | 5166 | 51.0 |  | 3674 | 51.3 | 1492 | 50.2 | χ^2^ = 73.8, p <0.001 |
|  | Vascular dementia | 2223 | 21.9 |  | 1609 | 22.5 | 614 | 20.7 |  |
|  | Lewy body dementia | 299 | 2.9 |  | 198 | 2.8 | 101 | 3.4 |  |
|  | Other dementia | 691 | 6.8 |  | 385 | 5.5 | 263 | 10.0 |  |
|  | Unspecified dementia | 1758 | 17.3 |  | 1291 | 18.0 | 467 | 15.7 |  |
| Year of diagnosis | 2008 | 1215 |  |  | 922 | 75.9 | 293 | 24.1 | χ^2^ = 113.9, p < 0.001 |
|  | 2009 | 1177 |  |  | 889 | 75.5 | 288 | 24.5 |  |
|  | 2010 | 1346 |  |  | 1020 | 75.8 | 326 | 24.2 |  |
|  | 2011 | 1445 |  |  | 1036 | 71.7 | 409 | 28.3 |  |
|  | 2012 | 1476 |  |  | 1021 | 69.2 | 455 | 30.8 |  |
|  | 2013 | 1545 |  |  | 1049 | 67.9 | 496 | 32.1 |  |
|  | 2014 | 1515 |  |  | 994 | 65.6 | 521 | 34.4 |  |
|  | 2015 | 418 |  |  | 236 | 56.5 | 182 | 43.5 |  |

**Key:** HoNOS = Health of the Nation Outcome Scale; MMSE = Mini-mental state examination

**Notes**: **^a^** Chi square test used to compare characteristics between admitted and non-admitted groups for categorical variables and t test used for continuous variables; ^b^ Based on clinical assessment nearest to first dementia diagnosis; ^c^ Figure for missing HoNOS score is for the HoNOS domain with most missing information.

Appendix 2: Distribution of count of general hospital admissions of people with dementia (n=10,137)

| **Count of admissions** | **During all follow-up** | | | | | |  | **During first year after diagnosis** | | | | | |
| --- | --- | --- | --- | --- | --- | --- | --- | --- | --- | --- | --- | --- | --- |
|  | All | | Emergency | | Elective | |  | All | | Emergency | | Elective | |
|  | n | % | n | % | n | % |  | n | % | n | % | n | % |
| 0 | 2444 | 24.1 | 2979 | 29.4 | 7532 | 74.3 |  | 5010 | 49.4 | 5708 | 56.3 | 8706 | 85.9 |
| 1 | 1919 | 18.9 | 2069 | 20.4 | 1396 | 13.8 |  | 2368 | 23.4 | 2184 | 21.5 | 976 | 9.6 |
| 2 | 1543 | 15.2 | 1560 | 15.4 | 591 | 5.8 |  | 1212 | 12.0 | 1071 | 10.6 | 277 | 2.7 |
| 3 | 1132 | 11.2 | 1072 | 10.6 | 244 | 2.4 |  | 657 | 6.5 | 506 | 5.0 | 93 | 0.9 |
| 4 | 798 | 7.9 | 709 | 7.0 | 141 | 1.4 |  | 353 | 3.5 | 285 | 2.8 | 27 | 0.3 |
| 5 | 554 | 5.5 | 482 | 4.8 | 67 | 0.7 |  | 194 | 1.9 | 156 | 1.5 | 16 | 0.2 |
| 6 | 423 | 4.2 | 327 | 3.2 | 50 | 0.5 |  | 132 | 1.3 | 87 | 0.9 | 6 | 0.1 |
| 7 | 308 | 3.0 | 225 | 2.2 | 21 | 0.2 |  | 77 | 0.8 | 56 | 0.6 | 4 | <0.1 |
| 8 | 214 | 2.1 | 165 | 1.6 | 14 | 0.1 |  | 28 | 0.3 | 28 | 0.3 | 4 | <0.1 |
| 9 | 157 | 1.5 | 117 | 1.2 | 12 | 0.1 |  | 28 | 0.3 | 15 | 0.1 | 4 | <0.1 |
| >10 | 645 | 6.4 | 432 | 4.3 | 69 | 0.7 |  | 78 | 0.8 | 41 | 0.4 | 24 | 0.2 |

Appendix 3: Predictors of general hospital admissions of people with dementia during all follow-up: multivariable negative binomial regression (n=7,863 with complete covariate information).

| Characteristic | | Emergency hospital admissions | | | |  | Elective hospital admissions | | |
| --- | --- | --- | --- | --- | --- | --- | --- | --- | --- |
|  |  | IRR (95% CI) | | | p-value |  | IRR (95% CI) | | p-value |
| Age (per 1 year increment) | | **1.03 (1.02, 1.03)** | | | **< 0.001** |  | **0.96 (0.95, 0.97)** | | **< 0.001** |
| Sex | Female | **0.74 (0.70, 0.79)** | | | **< 0.001** |  | **0.56 (0.49, 0.65)** | | **< 0.001** |
| Ethnicity | White (Ref) | 1 | |  | |  | 1 |  | |
|  | Asian | 0.87 (0.76, 1.00) | | | 0.05 |  | 1.07 (0.80, 1.43) | | 0.64 |
|  | African/Caribbean | **0.80 (0.73, 0.86)** | | | **< 0.001** |  | **1.71 (1.42, 2.06)** | | **< 0.001** |
|  | Other | **0.73 (0.62, 0.87)** | | | **< 0.001** |  | 1.31 (0.91, 1.87) | | 0.14 |
| Marital status | Married (Ref) | 1 |  | | |  | 1 |  | |
|  | Divorced | 1.11 (1.00, 1.24) | | | 0.05 |  | **1.49 (1.17, 1.91)** | | **0.001** |
|  | Widowed | 1.08 (1.00, 1.16) | | | 0.05 |  | 1.01 (0.86, 1.8) | | 0.94 |
|  | Single | 1.05 (0.97, 1.14) | | | 0.24 |  | 0.93 (0.77, 1.12) | | 0.45 |
| Deprivation score (per 10-unit increase in deprivation) | | **1.06 (1.04, 1.09)** | | | **< 0.001** |  | **0.93 (0.88, 0.99)** | | **0.02** |
| MMSE (per 1 unit decrease) | | 1.00 (1.00, 1.01) | | | 0.09 |  | **0.95 (0.94, 0.96)** | | **< 0.001** |
| Problem with  (from HoNOS subscale)^a^: | Agitated behaviour | 1.00 (0.93, 1.08) | | | 0.98 |  | 0.87 (0.72, 1.04) | | 0.13 |
|  | Self-injury | 1.31 (1.04, 1.65) | | | 0.02 |  | 0.68 (0.38, 1.20) | | 0.18 |
|  | Problem-drink/drugs | 1.04 (0.89, 1.22) | | | 0.60 |  | 1.27 (0.88, 1.82) | | 0.20 |
|  | Physical illness | **1.56 (1.46, 1.66)** | | | **< 0.001** |  | **1.79 (1.55, 2.06)** | | **<0.001** |
|  | Hallucinations | 0.98 (0.90, 1.07) | | | 0.68 |  | 0.79 (0.65, 0.95) | | 0.01 |
|  | Depressed mood | **1.05 (0.97, 1.14)** | | | **0.21** |  | **0.68 (0.56, 0.82)** | | **<0.001** |
|  | Daily living | **1.10 (1.03, 1.17)** | | | **0.004** |  | **1.30 (1.12, 1.51)** | | **0.001** |
|  | Living conditions | **1.12 (1.04, 1.23)** | | | **0.005** |  | 0.85 (0.70, 1.03) | | 0.10 |
| Last recorded dementia diagnosis | Alzheimer’s Disease (Ref) | 1 |  | | |  | 1 |  | |
|  | Vascular dementia | **1.42 (1.32, 1.53)** | | | **< 0.001** |  | **2.34 (1.96, 2.78)** | | **< 0.001** |
|  | Lewy body dementia | 1.25 (1.06, 1.47) | | | 0.008 |  | **1.98 (1.38, 2.82)** | | **<0.001** |
|  | Other dementia | 1.08 (0.96, 1.21) | | | 0.19 |  | **1.38 (1.07, 1.77)** | | **0.01** |
|  | Unspecified dementia | **1.44 (1.32, 1.57)** | | | **< 0.001** |  | **1.43 (1.17, 1.74)** | | **<0.001** |
| Year of diagnosis (per 1 year later) | 2008 (Ref) | 1 | | |  |  | 1 | |  |
|  | 2009 | 1.06 (0.95, 1.19) | | |  |  | 1.23 (0.95, 1.59) | |  |
|  | 2010 | 1.11 (0.99, 1.24) | | |  |  | **1.54 (1.20, 1.98)** | |  |
|  | 2011 | **1.13 (1.02, 1.26)** | | |  |  | **2.10 (1.66, 2.67)** | |  |
|  | 2012 | **1.12 (1.01, 1.25)** | | |  |  | **1.88 (1.48, 2.39)** | |  |
|  | 2013 | **1.15 (1.03, 1.28)** | | |  |  | **1.83 (1.43, 2.34)** | |  |
|  | 2014 | 1.05 (0.94, 1.17) | | |  |  | **1.79 (1.39, 2.30)** | |  |
|  | 2015 | 1.14 (0.95, 1.36) | | |  |  | 1.22 (0.82, 1.82) | |  |

Key: CI: Confidence Interval; HoNOS = Health of the nation outcome scales; IRR: Incidence rate ratio; MMSE = Mini-mental state examination

Notes: ^a^ HoNOS subscale, dichotomised to 0-1 (no or minor problem) and 2-4 (problem behaviour); Bold figures indicate p<0.05 in multivariable analysis

Appendix 4: Predictors of general hospital admissions during first year after dementia diagnosis; multivariable negative binomial regression using multiple imputation for missing covariates (n=10,137).

| Characteristic | | Emergency hospital admissions | | | |  | Elective hospital admissions | | |
| --- | --- | --- | --- | --- | --- | --- | --- | --- | --- |
|  |  | IRR (95% CI) | | | p-value |  | IRR (95% CI) | | p-value |
| Age (per 1 year increment) | | **1.03 (1.02, 1.03)** | | | **< 0.001** |  | **0.95 (0.94, 0.96)** | | **< 0.001** |
| Sex | Female | **0.77 (0.69, 0.80)** | | | **< 0.001** |  | **0.60 (0.50, 0.71)** | | **< 0.001** |
| Ethnicity | White (Ref) | 1 | |  | |  | 1 |  | |
|  | Asian | **0.81 (0.68, 0.96)** | | | **0.02** |  | 1.00 (0.71, 1.42) | | 0.99 |
|  | African/Caribbean | **0.83 (0.75, 0.91)** | | | **< 0.001** |  | **1.50 (1.20, 1.87)** | | **< 0.001** |
|  | Other | **0.75 (0.62, 0.92)** | | | **0.005** |  | 1.21 (0.57, 2.58) | | 0.38 |
| Marital status | Married (Ref) | 1 |  | | |  | 1 |  | |
|  | Divorced | 1.12 (0.97, 1.28) | | | 0.11 |  | 1.21 (0.77, 1.89) | | 0.38 |
|  | Widowed | **1.10 (1.01, 1.21)** | | | **0.03** |  | 0.97 (0.41, 1.28) | | 0.83 |
|  | Single | 1.10 (0.99, 1.23) | | | 0.08 |  | 0.90 (0.71, 1.14) | | 0.38 |
| Deprivation score (per 10-unit increase in deprivation) | | 1.03 (1.00, 1.06) | | | 0.06 |  | **0.92 (0.85, 0.98)** | | **0.01** |
| MMSE (per 1 unit decrease) | | 1.01 (1.00, 1.01) | | | 0.06 |  | **0.96 (0.95, 0.97)** | | **0.003** |
| Problem with  (from HoNOS subscale)^a^: | Agitated behaviour | 1.02 (0.93, 1.11) | | | 0.69 |  | 0.89 (0.71, 1.10) | | 0.29 |
|  | Self-injury | 1.23 (0.93, 1.61) | | | 0.15 |  | 0.61 (0.30, 1.23) | | 0.17 |
|  | Problem-drink/drugs | 1.15 (0.95, 1.39) | | | 0.16 |  | 1.15 (0.77, 1.73) | | 0.49 |
|  | Physical illness | **1.64 (1.51, 1.77)** | | | **< 0.001** |  | **1.67 (1.34, 2.09)** | | **<0.001** |
|  | Hallucinations | 1.00 (0.91, 1.11) | | | 0.97 |  | 0.93 (0.74, 1.16) | | 0.51 |
|  | Depressed mood | 1.09 (0.98, 1.20) | | | 0.10 |  | **0.66 (0.53, 0.82)** | | **< 0.001** |
|  | Daily living | **1.20 (1.11, 1.31)** | | | **< 0.001** |  | 1.25 (0.99, 1.56) | | 0.06 |
|  | Living conditions | **1.13 (1.01, 1.26)** | | | **0.03** |  | **0.79 (0.62, 0.99)** | | **0.04** |
| Last recorded dementia diagnosis | Alzheimer’s Disease (Ref) | 1 |  | | |  | 1 |  | |
|  | Vascular dementia | **1.43 (1.32, 1.56)** | | | **< 0.001** |  | **2.04 (1.67, 2.49)** | | **< 0.001** |
|  | Lewy body dementia | **1.25 (1.03, 1.52)** | | | **0.03** |  | 1.86 (1.20, 2.90) | | 0.006 |
|  | Other dementia | 1.07 (0.94, 1.23) | | | 0.31 |  | 1.02 (0.75, 1.39) | | 0.89 |
|  | Unspecified dementia | **1.42 (1.29, 1.56)** | | | **< 0.001** |  | **1.40 (1.12, 1.76)** | | **0.003** |
| Year of diagnosis (per 1 year later) | 2008 (Ref) | 1 | | |  |  | 1 | |  |
|  | 2009 | **1.18 (1.01, 1.38)** | | |  |  | 0.98 (0.72, 1.35) | |  |
|  | 2010 | **1.26 (1.08, 1.47)** | | |  |  | 1.26 (0.93, 1.70) | |  |
|  | 2011 | **1.21 (1.04, 1.41)** | | |  |  | **1.81 (1.36, 2.42)** | |  |
|  | 2012 | **1.32 (1.14, 1.53)** | | |  |  | **1.50 (1.12, 2.00)** | |  |
|  | 2013 | **1.28 (1.10, 1.48)** | | |  |  | **1.41 (1.05, 1.89)** | |  |
|  | 2014 | **1.29 (1.11, 1.50)** | | |  |  | **1.56 (1.15, 2.12)** | |  |
|  | 2015 | **1.39 (1.12, 1.73)** | | |  |  | 1.03 (0.67, 1.58) | |  |
|  | **Per year later** | **1.03 (1.01, 1.04)** | | | **0.002** |  | **1.06 (1.02, 1.10)** | | **0.003** |

Key: CI: Confidence Interval; HoNOS = Health of the nation outcome scales; IRR: Incidence rate ratio; MMSE = Mini-mental state examination

Notes: ^a^ HoNOS subscale, dichotomised to 0-1 (no or minor problem) and 2-4 (problem behaviour); Bold figures indicate p<0.05 in multivariable analysis
